# Molecular endotypes in sepsis: integration of multicohort transcriptomics based on RNA sequencing

**DOI:** 10.1186/s40560-025-00802-1

**Published:** 2025-05-30

**Authors:** Kengo Mekata, Michihito Kyo, Modong Tan, Nobuaki Shime, Nobuyuki Hirohashi

**Affiliations:** 1 Department of Radiation Disaster Medicine, Research Institute for Radiation Biology and Medicine, Hiroshima University, 1-2-3 Kasumi, Minami-ku, Hiroshima, 734-8551 Japan; 2 Department of Emergency and Critical Care Medicine, Graduate School of Biomedical and Health Sciences, Hiroshima University, Hiroshima, Japan; 3 IBM Japan Systems Engineering Co., Ltd, Tokyo, Japan

**Keywords:** Sepsis, Heterogeneity, Transcriptome, Gene, MRNA, Immune

## Abstract

**Background:**

The heterogeneity of host responses in sepsis has hindered efforts to develop targeted therapies for this large patient population. Although growing evidence has identified sepsis endotypes based on the microarray data, studies using RNA-seq data—which offers higher sensitivity and a broader dynamic range—remain limited. We hypothesized that integrating RNA-seq data from patients with sepsis would reveal molecular endotypes with distinct biological and clinical signatures.

**Methods:**

In this meta-analysis, we systematically searched for publicly available RNA-seq datasets of sepsis. Using identified datasets, we applied a consensus clustering algorithm to identify distinct endotypes. To characterize the biological differences between these endotypes, we performed gene-set enrichment analysis and immune cell deconvolution. Next, we investigated the association between these endotypes and mortality risks. We finally developed gene classifiers for endotype stratification and validated our endotype classification by applying these classifiers to an external cohort.

**Results:**

A total of 280 adults with sepsis from four datasets were included in this analysis. Using an unsupervised approach, we identified three distinct endotypes: coagulopathic (*n* = 83, 30%), inflammatory (*n* = 118, 42%), and adaptive endotype (*n* = 79, 28%). The coagulopathic endotype exhibited upregulated coagulation signaling, along with an increased monocyte and neutrophil composition, although the adaptive endotype demonstrated enhanced adaptive immune cell responses, marked by elevated T and B cell compositions. The inflammatory endotype was characterized by upregulated TNF-α/NF-κB signaling and IL-6/JAK/STAT3 pathways with an increased neutrophil composition. Patients with the coagulopathic endotype had a significantly higher risk of mortality than those with the adaptive endotype (30% vs. 16%, odds ratio 2.19, 95% confidence interval 1.04–4.78, *p* = 0.04). To enable the practical application of these findings, we developed endotype classification models and identified 14 gene classifiers. In a validation cohort of 123 patients, we consistently identified these three endotypes. Furthermore, the mortality risk pattern was reproduced, with the coagulopathic endotype showing greater mortality risk than the adaptive endotype (34% vs 18%, *p* = 0.10).

**Conclusions:**

This multicohort RNA-seq meta-analysis identified three biologically and clinically distinct sepsis endotypes characterized by coagulopathic, adaptive, and inflammatory responses. This endotype-based approach to patient stratification may facilitate the development of more precise therapeutic strategies for sepsis.

**Supplementary Information:**

The online version contains supplementary material available at 10.1186/s40560-025-00802-1.

## Background

Sepsis is a life-threatening disease caused by the dysregulation of the host immune response to infection. Despite its high morbidity and mortality rates, leading to 11 million deaths annually worldwide [1], effective definitive therapy for sepsis has yet to be developed, in part due to its highly heterogeneous nature [2]. Although sepsis has been considered a single disease entity, growing evidence reveals significant heterogeneity in clinical presentations and outcomes [3–5]. Furthermore, emerging evidence using transcriptomic data indicates that sepsis has distinct molecular endotypes—subtypes with different underlying biological mechanisms [6–10].

Several microarray-based studies have identified sepsis endotypes that are clinically and biologically distinct [6–8]. However, these endotypes identified across different studies show some discrepancies, potentially due to variations in patient populations and technical limitations. The limited sensitivity and dynamic range of microarray may hinder the detection of subtle transcriptomic differences that are clinically meaningful. In contrast, RNA-seq enables a more comprehensive and highly sensitive profiling of gene expression [11, 12], potentially uncovering biologically distinct patient subtypes that may not be detectable by previous technologies. Despite these clinically relevant advantages, approaches using RNA-seq for sepsis endotyping remain limited [10]. Moreover, no study has systematically integrated diverse RNA-seq datasets to account for genetic and environmental variations in sepsis presentation.

To address this knowledge gap, we integrated and analyzed publicly available RNA-seq data from adult patients with sepsis to (1) identify endotypes based on the RNA-seq data, (2) clarify the biological characteristics of these endotypes, (3) examine the clinical outcomes associated with the endotypes, and (4) develop gene classifiers for endotype identification. We hypothesized that this RNA-seq-based approach would reveal molecular endotypes with distinct biological and clinical signatures.

## Methods

### Study design and analytic workflow

We conducted a meta-analysis using publicly available RNA-seq datasets to identify endotypes in sepsis. The analytic workflow is summarized in Figure S1. Briefly, we first performed a systematic search to identify eligible RNA-seq data of sepsis. To ensure robust analysis, we preprocessed the data by excluding low-quality samples (e.g., those with low mapping rates), filtering out low-expression genes, performing normalization, and correcting for batch effects across datasets. Second, we applied an unsupervised clustering approach to preprocessed RNA-seq data to identify distinct molecular endotypes. Third, to interpret the biological significance of the endotypes, we performed differential expression gene analysis, pathway enrichment analysis, and immune cell deconvolution analysis. Fourth, we investigated the clinical characteristics and mortality associated with each endotype to assess their potential clinical relevance. Finally, we constructed a multiclass regression model with LASSO regularization to identify gene classifiers for endotype determination. To validate the robustness of our findings, we applied these classifiers to an independent external cohort and assessed the reproducibility of the endotypes and their associated characteristics.

### Data acquisition

To systematically acquire mRNA-seq data from patients diagnosed with sepsis in public databases, we searched MEDLINE, Scopus, and Web of Science published before April 1st, 2024. The detailed search strategy can be found in Table S1. Two independent reviewers (KM and MK) assessed whether the studies satisfied the inclusion criteria. The inclusion criteria were: (1) adult patients diagnosed with sepsis, (2) mRNA-seq data obtained from human whole blood, and (3) available mortality outcome data. Studies were excluded if they met the following criteria: (1) Meta-analyses, systematic reviews, case reports, and conference abstracts, (2) Studies using microarray or single-cell RNA-seq data, (3) Studies primarily focusing on COVID-19, and (4) Studies using publicly available data. Any disagreements were resolved through discussion between the reviewers. From the identified studies, we included data that met the following criteria: (1) Patients meeting Sepsis-3 criteria, with three exceptions: (i) for GSE63042 [13] and GSE131411 [14] using Sepsis-2 definitions, we included patients with severe sepsis or septic shock to ensure alignment with current Sepsis-3 criteria; (ii) for GSE185263 [15], which included patients with suspected sepsis, we selected only those with sequential organ failure assessment (SOFA) scores ≥ 2 to ensure they met the Sepsis-3 diagnostic criteria; (iii) for GSE222393 [16], which included some patients overlapping with GSE185263, we included only unique patients based on the metadata, and (2) For longitudinal studies, we analyzed only the initial time point data (collected within 24 h of emergency department [ED] or intensive care unit [ICU] admission). This analysis was exempt from IRB review as it utilized de-identified, publicly available data.

### RNA-seq data preprocessing

We obtained RNA-seq data in sequence read archive (SRA) format and converted it to FASTQ files using the SRA Toolkit [17]. We used Fastp to remove low-quality reads and trim adapters for quality control of the raw sequencing data [18]. We quantified the processed files using Salmon with the human transcriptome (GRCh38) as a reference, with GC bias correction (-gcBias) and mapping validation (-validateMappings) options [19]. We analyzed the count data using the R *tximport* package to convert transcript levels to gene-level expression [20]. We excluded samples with mapping rates below 60% to ensure high-quality gene expression data [21].

We filtered low-expression genes using the filterByExpr function from the R *edgeR* package [22]. We normalized the filtered count data using the trimmed mean of M values method and corrected for batch effects among different datasets using the ComBat-seq function from the R *sva* package [23, 24].

### Endotyping with RNA-seq data

We aimed to identify distinct endotypes based on thegene expression without prior assumptions. To achieve this, we computed a distance matrix and derived mutually exclusive clusters using a consensus clustering algorithm in the R *ConsensusClusterPlus* package [25]. The algorithm was run with 100 resampling iterations, using 80% subsampling of samples and 100% of features per iteration, with k-means clustering and Euclidean distance. We determined the optimal number of clusters by evaluating three metrics: consensus matrices, cluster consensus values, and the relative change in area under the cumulative distribution function (CDF) curve.

### Biological significance of endotypes

To interpret the biological differences between the endotypes, we performed three analyses. First, we conducted differential expression gene (DEG) analysis between the endotypes using the R *limma* package [26]. We defined DEGs as genes with absolute Log_2_ fold change ≥ 1 and false discovery rate (FDR) < 0.05. Second, we performed Gene Set Enrichment Analysis (GSEA) using the R *fgsea* package based on the Molecular Signatures Database Hallmark and Gene Ontology biological process gene sets [27–29]. Third, we estimated the proportions of immune cells in each sample using the CIBERSORTx [30]. We input transcripts per million-normalized gene expression data and calculated immune cell abundance using the LM22 signature matrix [31], which quantifies 22 types of immune cells.

### Mortality of endotypes

To determine the association of the endotypes with mortality, we constructed an unadjusted logistic regression model.

### Identifying endotype classifier and validation in an external dataset

To identify a gene classifier for endotype determination, we constructed a multiclass regression model with the least absolute shrinkage and selection operator (LASSO) regularization using the R *caret* package. We applied fivefold cross-validation to minimize potential overfitting and selected the top 200 DEGs for each cluster comparison based on FDR as input. We determined the gene classifiers based on importance scores in the model. We then validated our endotype classification using an external cohort (GSE236713 [32]) by applying the consensus clustering algorithm to the expression of the gene classifier from microarray data. GSE236713 dataset contains microarray data from adult patients with sepsis. This dataset was chosen because it provides comprehensive patient data including laboratory data and mortality outcomes, allowing us to validate both the biological characteristics and clinical relevance of our identified endotypes. Unlike the discovery cohort, GSE236713 was not identified through our systematic search but was selected based on its availability of detailed clinical data supporting our analysis objectives. We assessed the consistency of the endotypes by comparing their GSEA patterns and clinical characteristics with those of the original cohort.

### Statistical analysis

We analyzed the data using R version 4.4.1 (R foundation, Vienna, Austria). All *P* values were two-tailed, with *p* < 0.05 considered statistically significant. We used chi-square and Kruskal–Wallis tests, as appropriate, to compare clinical characteristics and immune cell proportions between the endotypes. For variables showing significant differences, we conducted pairwise comparisons using Wilcoxon tests with Bonferroni correction for multiple testing. Confidence intervals for median differences were estimated using bootstrapping with 1,000 iterations. We accounted for multiple testing using the Benjamini–Hochberg FDR method to the RNA-seq data, which allows for the interpretation of statistical significance in the context of multiple hypothesis testing [33].

## Results

### Literature search and study and patient selection

**Fig. 1:**
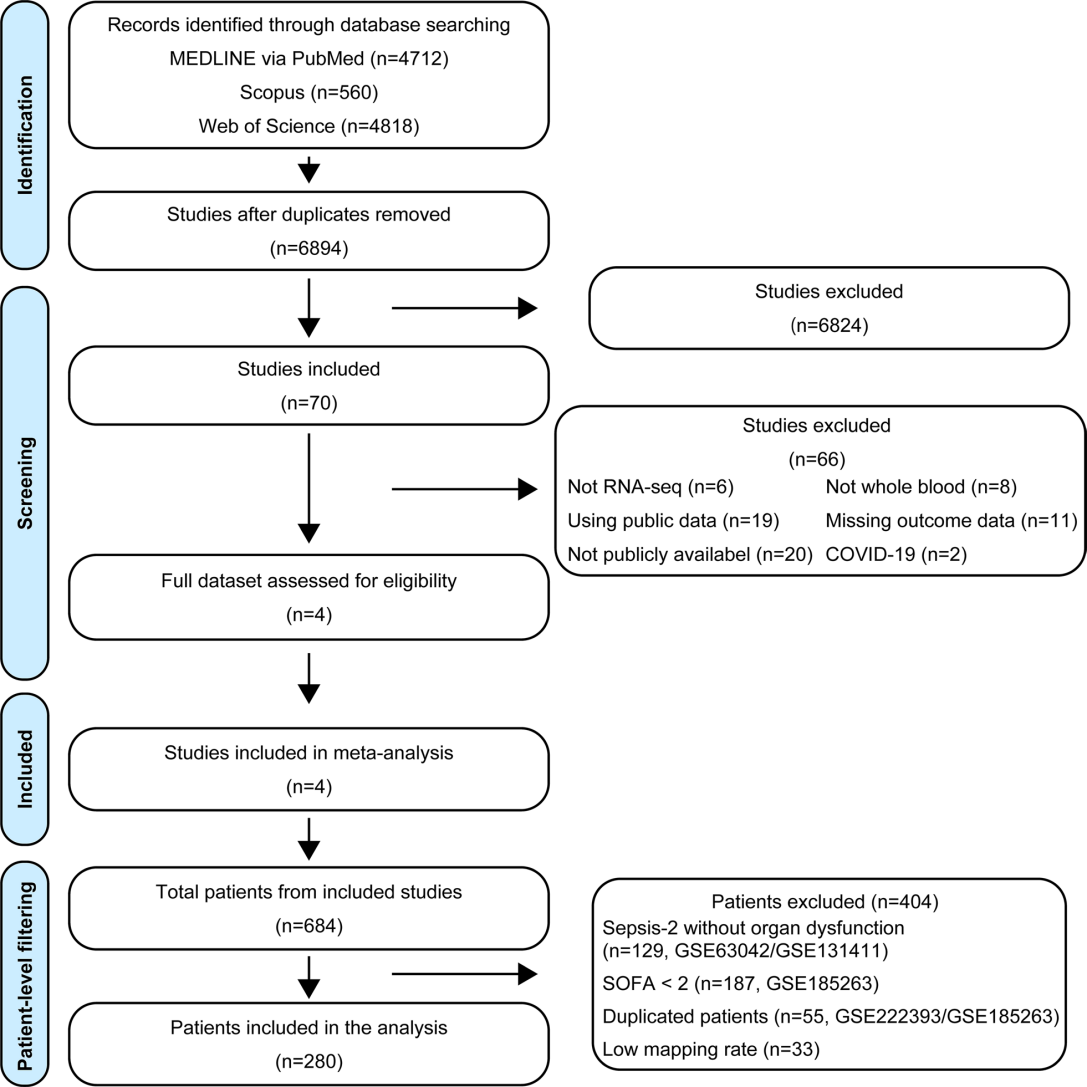
Flowchart of study and patient selection. To acquire mRNA-seq data from patients with sepsis, we systematically searched MEDLINE, Scopus, and Web of Science databases. We initially identified 6,894 studies after removing duplicates. Title and abstract screening resulted in the selection of 70 studies for full-text and metadata assessment. Of these, four studies met all eligibility criteria and were included in the meta-analysis. At the patient level, we finally included 280 patients in this analysis after excluding 404 patients

We reviewed 6,894 records after the removal of duplicates. Following the initial screening, we evaluated the full-text and metadata of 70 articles for eligibility. Of these, four studies [13–16] met all inclusion criteria and were included in this meta-analysis (Fig. 1). Among the 684 patients from these studies, we included 280 patients in the final analysis after excluding 404 patients according to predefined criteria.

### Patient characteristics

After preprocessing, we analyzed data from a total of 280 adult patients with sepsis (Table 1). In GSE63042, only patients with severe sepsis and septic shock, as defined by the Sepsis-2 criteria, were included. All blood samples for RNA-seq were collected within 24 h of admission to the ED or ICU across all studies. The included studies represented diverse geographical populations: North America (United States, Canada), Europe (Switzerland, the Netherlands), South America (Colombia), and Oceania (Australia).

**Table 1:** Datasets included in the study

Accession number	Publish year	First author	Description of patients	Timing of collecting sample	Sample size (N)	Criteria for sepsis	Mortality (percent)	Country
GSE63042 [13]	2014	Tsalik	Adults in ED with severe sepsis or septic shock	At ED presentation	64	Sepsis-2	38	USA
GSE131411 [14]	2019	Braga	Adults in ICU with septic shock	Within 16 h of ICU admission	14	Sepsis-2	20	Switzerland
GSE185263 [15]	2022	Baghela	Adults in ED or ICU with sepsis	Within 2 h of ED admission or within the first day of ICU admission	199	Sepsis-3	14	Australia Canada Colombia Netherlands
GSE222393 [16]	2023	An	Adults in ICU with sepsis	Within 24 h of ICU admission	3	Sepsis-3	0	Canada

ED, emergency department; ICU, intensive care unit; SIRS, systemic inflammatory response syndrome

### Identification of three distinct endotypes

**Fig. 2:**
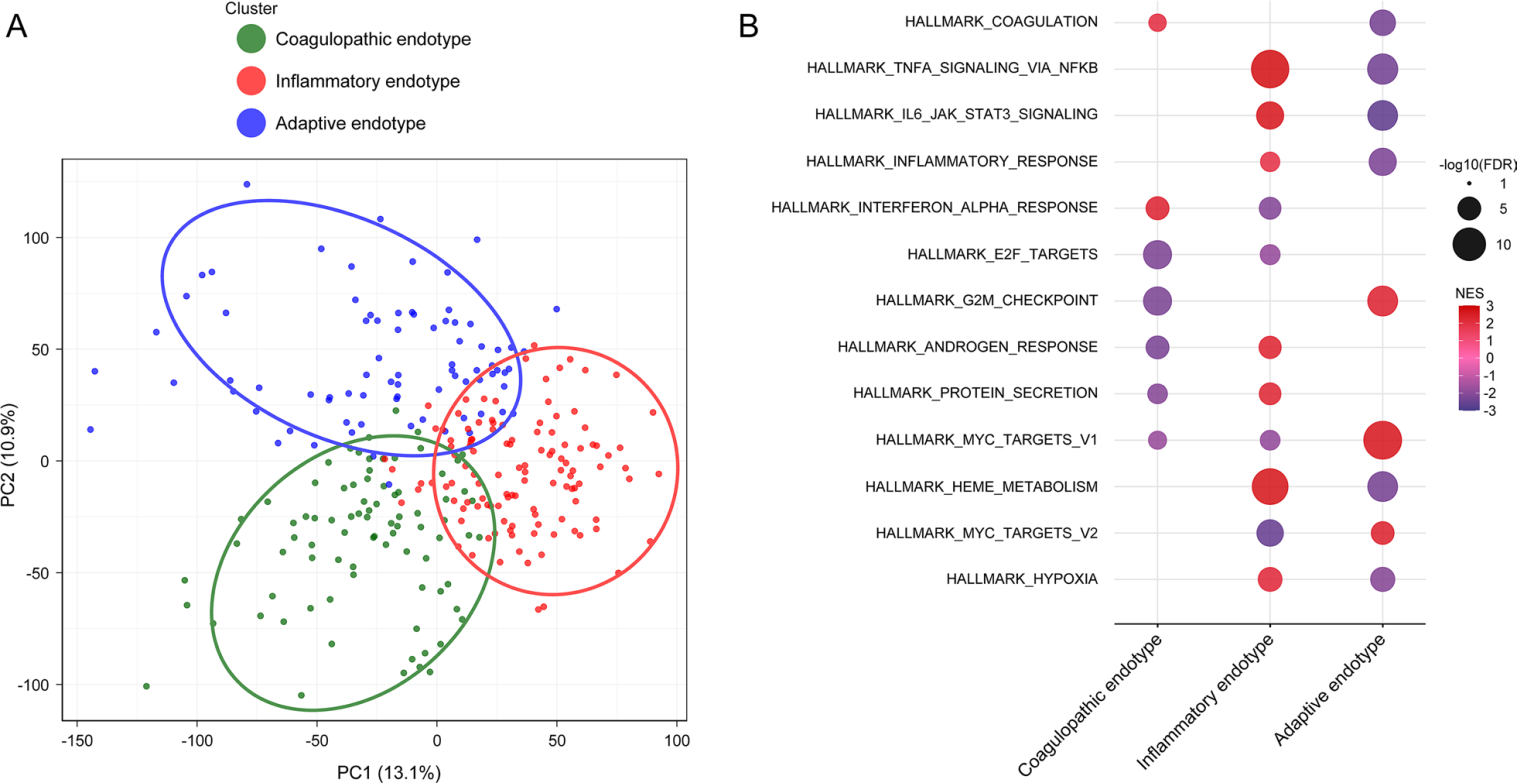
Distinct molecular signatures and enriched biological pathways across endotypes. (**A**) Principal component analysis (PCA) of transcriptomic profiles corresponding to endotypes. The PCA plot shows the distribution of patient samples in a low-dimensional space, where each dot represents an individual patient's mRNA signature. The three endotypes are indicated by different colors: coagulopathic (green), inflammatory (red), and adaptive (blue) endotype. **(B) **Gene Set Enrichment Analysis (GSEA) forspecificbiological pathways across endotypes. GSEA was performed based on the Molecular Signatures Database (MSigDB) Hallmark gene sets. Significantly enriched (FDR < 0.05) and high NES (NES > 2) pathways in any endotypes are listed. The dots size represents FDR (-log10 scale). The color gradient indicates the normalized enrichment score (NES), indicating the degree and direction of pathway enrichment relative to the endotype. FDR, false discovery rate; MSigDB, molecular signatures database; NES, normalized enrichment score; PCA, principal component analysis

We conducted preprocessing of RNA-seq data, including normalization and batch correction to minimize potential study-specific effects. Principal component analysis (PCA) plots demonstrated a successful reduction of study-specific sample clustering following batch correction (Figure S2). After quality filtering, a total of 14,858 genes were retained for further clustering analysis. By applying a consensus clustering approach to the transcriptomic data, the combination of the consensus matrix, cluster consensus value, and relative change in the area under the CDF curve identified that a three-class model provided an optimal fit (Figure S3). Based on the subsequent GSEA and immune cell deconvolution, we designated these three endotypes as coagulopathic, inflammatory, and adaptive endotypes. The PCA plot showed distinct transcriptional profiles corresponding to these three endotypes (Fig. 2A).

### Distinct biological function of endotypes

To investigate the biological significance of the endotypes, we performed GSEA. GSEA revealed distinct biological pathways characterizing each endotype (FDR < 0.05; Fig. 2B; Tables S2 and S3). The coagulopathic endotype was characterized by upregulated coagulation-related pathways (e.g., coagulation, regulation of angiogenesis) and interferon (IFN)-α pathway. The inflammatory endotype exhibited upregulated inflammatory pathways (e.g., tumor necrosis factor [TNF]-α/nuclear factor kappa B [NFκB] signaling, interleukin [IL]−6/Janus kinase [JAK]/signal transducer and activator of transcription [STAT] 3 signaling, cytokine-mediated signaling). The adaptive endotype demonstrated upregulated Myc-related pathways and adaptive immune responses (e.g., adaptive immune response, T cell-related pathway).

**Fig. 3:**
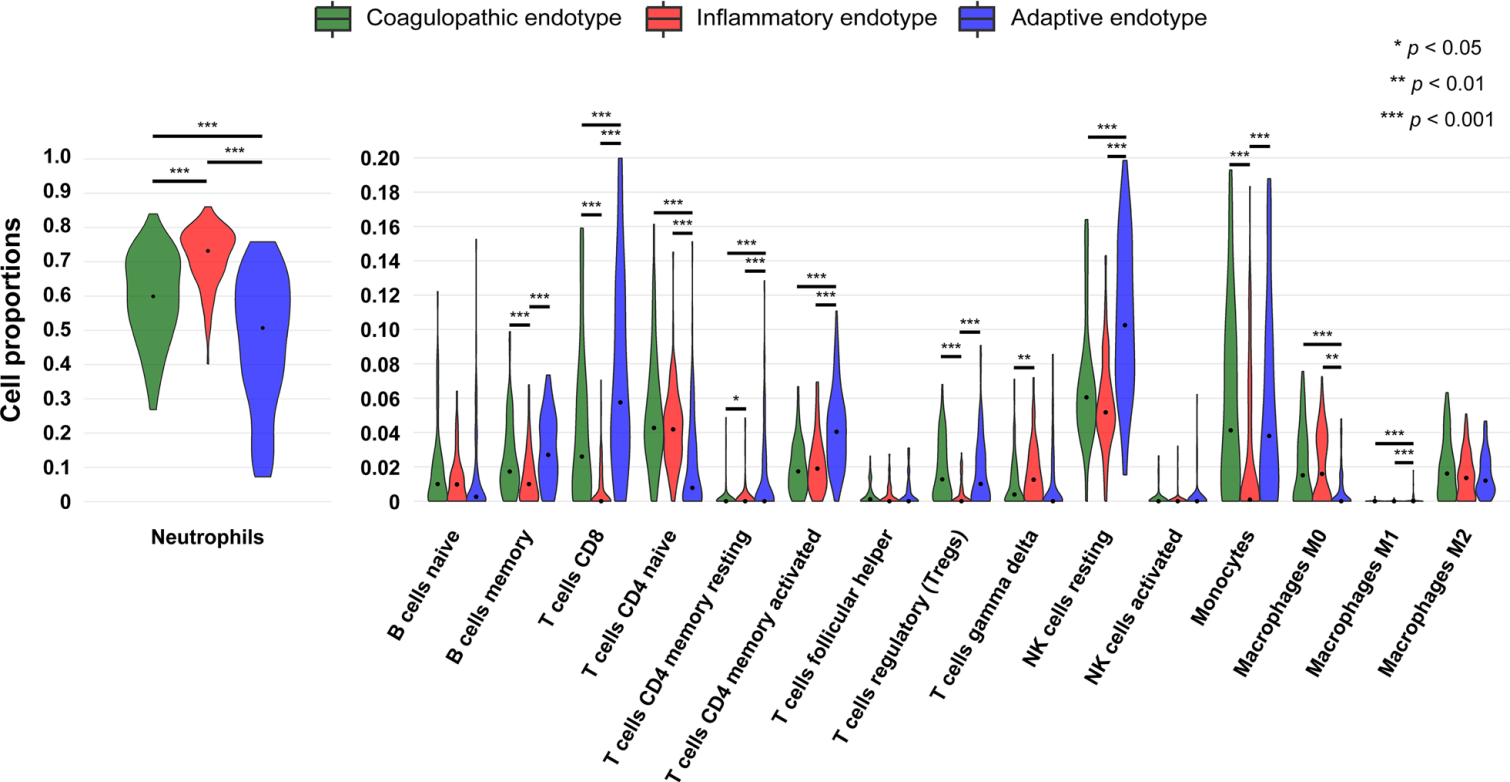
Immune cell composition across endotypes. To estimate the proportions of immune cells in each endotype, we used CIBERSORTx. The violin plots represent the distribution of immune cell proportions (*y* axis) for each cell type across endotypes. Statistical differences were assessed using Kruskal–Wallis tests followed by Bonferroni-corrected post hoc tests for cell types showing significant differences. Asterisks indicate statistical significance: ***p < 0.001, **p < 0.01, *p < 0.05. NK, natural killer

Next, to examine the immune cell type composition of each endotype, we performed transcriptome deconvolution analysis of the RNA-seq data. The coagulopathic endotype demonstrated a significantly higher composition of monocytes and regulatory T cells (Treg) than the inflammatory endotype (median difference 4.2%, 95% confidence interval [CI] 2.7–7.1, *p* < 0.001; and 1.3%, 95% CI 0.6–1.9, *p* < 0.001, respectively), and a significantly higher composition of neutrophils than the adaptive endotype (median difference 9.2%, 95% CI 3.5–19.3, *p* < 0.001; Figs. 3 and S4; Table S4). The inflammatory endotype was characterized by a significantly higher composition of neutrophils compared to both the coagulopathic (median difference 13.3%, 95% CI 8.3–17.6, *p* < 0.001) and adaptive endotypes (median difference 22.5%, 95% CI 17.8–31.1, *p* < 0.001). In contrast, the adaptive endotype showed a significantly higher composition of adaptive immune cells, including CD8^+^ T cells (vs. coagulopathic endotype: median difference 4.4%, 95% CI 1.2–7.2, *p* < 0.001; vs. inflammatory endotype: median difference 7.0%, 95% CI 5.0–9.4, *p* < 0.001) and activated memory CD4^+^ T cells (vs. coagulopathic endotype: median difference 2.3%, 95% CI 1.4–3.1, *p* < 0.001; vs. inflammatory endotype: median difference 2.2%, 95% CI 1.2–2.9, *p* < 0.001).

### Clinical characteristics of endotypes

**Fig. 4:**
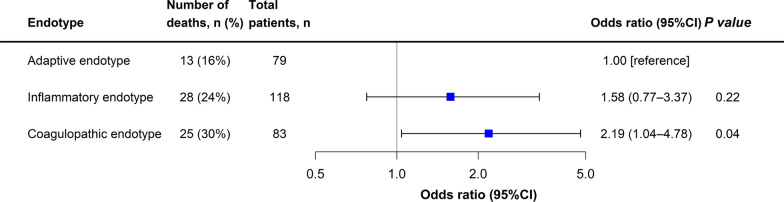
Association of endotypes with mortality. To investigate the association of endotypes (Adaptive endotype as the reference) with mortality, logistic regression model was constructed. The forest plot displays odds ratios with 95% confidence intervals (CI) for mortality across the endotypes. CI, confidence interval

Age differed significantly across endotypes (*p* = 0.003; Table S5). Patients with the coagulopathic and inflammatory endotypes were older than those with the adaptive endotype (median difference 12 years, 95% CI 0–24, *p* = 0.01; and 10 years, 95% CI 2–20, *p* = 0.01, respectively; Table S6). SOFA scores also differed significantly across endotypes (*p* < 0.001). Patients with the inflammatory endotype had higher SOFA scores than those with the coagulopathic and adaptive endotypes. (median difference 2, 95% CI 1–4, *p* = 0.0002; and 2, 95% CI 1–4, *p* = 0.001, respectively). In the logistic regression model, patients with the coagulopathic endotype had significantly higher mortality compared to those with the adaptive endotype (30% vs. 16%, odds ratio 2.19, 95% CI 1.04–4.78, *p* = 0.04; Fig. 4).

### Development of a gene classifier for endotype classification and validation in an external dataset

Differential expression analysis identified 1,176 DEGs in the coagulopathic endotype, 714 DEGs in the inflammatory endotype, and 1,434 DEGs in the adaptive endotype (Figure S5). By applying a multiclass regression model with LASSO regularization using the top 200 DEGs from each comparison, we identified a 14-gene classifier that achieved 94% accuracy in endotype classification (Table S7). We then validated our endotypes by applying the 14-gene classifier to microarray data from the validation dataset (GSE236713) using a consensus cluster algorithm. The three-class model provided an optimal fit (Figure S6). GSEA confirmed that these endotypes aligned with our previously identified coagulopathic, inflammatory, and adaptive endotypes (Tables S8 and S9). Among clinical parameters, platelet counts differed significantly across endotypes, with the coagulopathic endotype demonstrating the lowest median counts (163 × 10^9^/L, IQR 115–259) compared to inflammatory (221 × 10^9^/L, IQR 170–341) and adaptive endotypes (215 × 10^9^/L, IQR 154–302) (overall *p* = 0.04; Table 2). Patients with the coagulopathic endotype showed a trend toward higher mortality compared to those with the adaptive endotype (34% vs. 18%, odds ratio 2.36, 95% CI 0.87–6.95, *p* = 0.10; Tables 2 and S10).

**Table 2:** Characteristics of patients in each endotype in the validation cohort

	**Coagulopathic endotype** **(n = 44)**	**Inflammatory** **endotype** **(n = 40)**	**Adaptive** **endotype** **(n = 39)**	***P* value**	**Total n**^†^
Age, (year), median (IQR)	71 (64–79) (n = 44)	68 (61–74) (n = 40)	67 (52–74) (n = 38)	0.17	122
Male sex, n (%)	22 (50) (n = 44)	23 (59) (n = 39)	17 (45) (n = 38)	0.48	121
APACHE II score, median (IQR)	32 (22–39) (n = 42)	32 (25–40) (n = 37)	29 (18–35) (n = 33)	0.28	112
White blood cells, (10^3^/µL), median (IQR)	15.7 (11.8–22.8) (n = 44)	16.1 (11.0–20.7) (n = 40)	15.0 (10.5–18.9) (n = 39)	0.74	123
Neutrophils, (10^3^/µL), median (IQR)	14.0 (9.3–20.5) (n = 44)	14.9 (8.9–18.6) (n = 40)	12.4 (8.7–16.4) (n = 39)	0.63	123
Lymphocytes, (10^3^/µL), median (IQR)	0.6 (0.4–1.1) (n = 44)	0.7 (0.5–1.0) (n = 40)	0.7 (0.4–1.3) (n = 39)	0.85	123
Platelets, (10^9^/L), median (IQR)	163 (115–259) (n = 44)	221 (170–341) (n = 40)	215 (154–302) (n = 39)	0.04	123
CRP, (mg/L), median (IQR)	223 (158–314) (n = 42)	201 (109–325) (n = 40)	244 (92–294) (n = 38)	0.55	120
Mortality, n (%)	15 (34) (n = 44)	8 (20) (n = 40)	7 (18) (n = 39)	0.19	123

^†^ Sample sizes vary across variables due to missing data

Statistical comparisons between endotypes were performed using chi-square and Kruskal–Wallis tests as appropriate

APACHE, acute physiology and chronic health evaluation; CRP, C-reactive protein; IQR, interquartile range

## Discussion

Based on the current analysis of RNA-seq data from four studies comprising 280 patients with sepsis, we identified three biologically and clinically distinct transcriptomic endotypes. The coagulopathic endotype was characterized by upregulated coagulation pathways with higher monocyte, Treg, and neutrophil composition. The inflammatory endotype showed higher SOFA scores, with upregulated TNF-α/NFκB and IL-6/JAK/STAT3 signaling pathways. In contrast, the adaptive endotype was characterized by younger age and upregulated pathways related to the adaptive immune response with higher composition of adaptive immune cells. Notably, the coagulopathic endotype demonstrated significantly higher mortality than the adaptive immune endotype. To the best of our knowledge, this is the first investigation that systematically integrated multicohort RNA-seq data to identify biologically and clinically distinct transcriptomic endotypes in patients with sepsis.

The emerging evidence, consistent with our findings, suggests that sepsis has biological and clinical heterogeneity based on transcriptome data. A previous multicenter study using microarray-based transcriptome data from patients with sepsis due to pneumonia has revealed that sepsis has two biologically distinct clusters, SRS 1 and 2. SRS 1 was characterized by relative immunosuppression, endotoxin tolerance, T-cell exhaustion, and metabolic derangement, which were associated with a worse prognosis [7]. Another previous multicenter study using microarray-based transcriptome data from patients with sepsis has also reported that sepsis has four biologically and clinically distinct clusters (MARS 1–4) [8]. In addition, a previous meta-analysis integrating microarray-based transcriptomic data from 13 studies has reported that sepsis has three endotypes (coagulative, inflammopathic, and adaptive subtype) [6]. The coagulative and inflammopathic subtypes had worse outcomes than the adaptive subtype. Although different endotypes have been identified across previous studies, possibly due to the heterogeneity of sepsis etiology and patient characteristics, our findings were consistent with the previously reported three-endotype model (coagulative, inflammopathic, and adaptive subtype). Importantly, we validated the robustness of this three-endotype model and identified novel classifier genes that were distinct from previously identified classifiers [6] using RNA-seq analysis across multiple cohorts. These findings not only build upon previous endotyping efforts but also provide deeper insights into the molecular heterogeneity of sepsis responses, highlighting the potential for more precise therapeutic targeting.

The exact mechanisms underlying the observed endotypes—particularly the coagulopathic endotype associated with the highest risk of mortality—warrant clarification. In alignment with our findings about the coagulopathic endotype, a previous endotyping study based on mRNA-seq data has shown that the high-mortality subtype was characterized by an enhanced coagulation pathway [10]. In addition, our observation of higher composition of monocytes and neutrophils suggested a potential exacerbation of immunothrombosis, where these innate immune cells actively contribute to pathological coagulation through proinflammatory cytokines produced by monocytes and neutrophil extracellular trap (NET) formation [34]. At the molecular level, type I interferon, particularly IFN-α, promotes coagulation through enhanced high mobility group box 1 production, which subsequently activates tissue factor expression and platelet aggregation [35]. In addition, the simultaneous upregulation of angiogenic pathways suggests crosstalk between vascular dysfunction and coagulation activation in disseminated intravascular coagulopathy [36]. Our findings warrant the development of definitive therapy for this endotype that has an intricate relationship between inflammation and coagulation in sepsis. In contrast to the coagulopathic endotype, the adaptive endotype represents a well-regulated immune response characterized by the coordinated activation of Myc-dependent and enhanced adaptive immune responses. At the molecular level, T cell antigen receptor engagement triggers multiple downstream signaling cascades, including the activation of Myc [37], which acts as a key regulator of lymphocyte proliferation and metabolism [38]. Importantly, this endotype demonstrates tissue damage prevention (lower SOFA scores) and improved survival rates, achieved through the coordinated upregulation of adaptive immune components without excessive inflammatory cytokine production. The inflammatory endotype had a higher risk of mortality than the adaptive endotype although statistically insignificant. Beyond our observation of a well-established upregulation of TNF-α/NFκB and IL-6/JAK/STAT3 signaling pathways indicating hyperinflammation, we identified a significant increase in neutrophil composition, suggesting that neutrophils play a more significant role in inflammation. The expanded neutrophil population could amplify inflammatory cascades not only through direct cytokine production but also via suppression of CD4^+^ T cell [39], potentially exacerbating the progression of inflammation. Although previous clinical trials of TNF-α monoclonal antibodies have not shown overall benefit in unselected sepsis populations [40], our findings suggest that patient stratification into the inflammatory endotype might identify a subgroup more likely to benefit from these targeted therapies.

This study has several potential limitations. First, the criteria for sepsis varied across studies. Although we included patients who met the definitions of severe sepsis or septic shock according to the Sepsis-2 criteria in GSE63042, some patients may not have satisfied the Sepsis-3 criteria. Second, our endotypes were derived from transcriptomic data obtained at a single time point. Nevertheless, the findings of this study during the early course of sepsis provide biologically and clinically significant insights. Third, the timing of mortality assessment differed across studies. However, significant differences between endotypes remained when mortality was compared at the same time point in the validation cohort. Fourth, the discovery and validation cohorts differed in disease severity. The discovery cohort had a median SOFA score of 3–5, although the validation cohort had a median APACHE II score of 29–32, indicating substantially higher severity. Despite this difference, the three-endotype model and their clinical and biological patterns were consistent in both cohorts, suggesting the robustness of our results. However, this discrepancy in cohort characteristics warrants further validation studies with more balanced severity distributions to confirm the generalizability of our findings. Lastly, detailed clinical characteristics were unavailable for the discovery cohort, precluding comprehensive clinical comparisons in this analysis. Nevertheless, we identified significant differences in detailed clinical characteristics across endotypes in the validation cohort.

## Conclusion

In conclusion, by applying an unsupervised clustering approach to integrated RNA-seq data from four studies of sepsis, we identified three biologically and clinically distinct transcriptomic endotypes. Specifically, the coagulopathic endotype—characterized by upregulated coagulation pathways with higher monocyte, Treg, and neutrophil composition—demonstrated significantly higher mortality. In addition, we developed a novel 14-gene classifier that enables practical endotype identification in patients with sepsis. The robust identification of these endotypes not only advances our understanding of the diverse biological mechanisms underlying sepsis but also provides a framework for developing targeted therapies for sepsis with a substantial morbidity and mortality burden.

## Supplementary Information

Supplementary Material 1

Supplementary Material 2

Supplementary Material 3

Supplementary Material 4

Supplementary Material 5

Supplementary Material 6

## Data Availability

The gene expression datasets used in this study are publicly available in the Gene Expression Omnibus database under the accession numbers GSE63042, GSE131411, GSE185263, and GSE222393 and in the Sequence Read Archive (SRA) database under the accession number SRP339377. R codes used for the analyses are available on GitHub (https://github.com/K-Mekata-bio/Sepsis-RNA-seq-Endotype).

## Abbreviations

CDF: Cumulative distribution function
CI: Confidence interval
DEG: Differential expression gene
ER: Emergency department
FDR: False discovery rate
GSEA: Gene set enrichment analysis
ICU: Intensive care unit
IL: Interleukin
JAK: Janus kinase
LASSO: Least absolute shrinkage and selection operator
NF-κB: Nuclear factor kappa B
PCA: Principal component analysis
RNA-seq: RNA sequencing
SOFA: Sequential organ failure assessment
SRA: Sequence read archive
STAT: Signal transducer and activator of transcription
TNF: Tumor necrosis factor
Treg: Regulatory T cell
