# Supplementary material for: Molecular endotypes in sepsis: integration of multicohort transcriptomics based on RNA sequencing
Source: J Intensive Care. 2025 May 30;13:30. doi: 10.1186/s40560-025-00802-1 (PMC12123803; doi:10.1186/s40560-025-00802-1)
Supplement: Supplementary file 1 — Supplementary Material 1 [file 40560_2025_802_MOESM1_ESM.docx]

**Supplementary Materials**

**Title:** Molecular endotypes in sepsis: Integration of multi-cohort transcriptomics based on RNA sequencing

**Authors:** Kengo Mekata, MMSc^*^; Michihito Kyo, MD, PhD^*^; Modong Tan, PhD; Nobuaki Shime, MD, PhD; Nobuyuki Hirohashi, MD, PhD

* These authors have contributed equally and are designated to have co-first authorship

**Table of contents**

**Supplementary Methods**

**Supplementary References**

**Table S1.** Search strategies

**Table S2.** Significantly enriched Hallmark biological pathways across endotypes

**Table S3.** Significantly enriched GO biological processes across endotypes

**Table S4.** Pairwise comparisons of immune cells across endotypes

**Table S5.** Characteristics of patients in each endotype

**Table S6.** Pairwise comparisons of age and SOFA score across endotypes

**Table S7.** Endotype assignment matrix and gene importance scores for endotype classification

**Table S8.** Significantly enriched biological pathways based on MSigDB Hallmark gene sets in the validation dataset

**Table S9.** Significantly enriched biological pathways based on MSigDB Gene Ontology biological process gene sets in the validation dataset

**Table S10.** Association of endotypes with mortality in the validation cohort

**Table S11.** List of tools and package used in this analysis

**Figure S1.** Study design and analytical workflow for sepsis endotyping

**Figure S2.** Principal component analysis plots before and after batch correction

**Figure S3.** Consensus matrices, cluster consensus values, and CDF plot according to the number of clusters

**Figure S4.** Immune cell composition of immune cells with lower cell proportion across endotypes

**Figure S5.** Differential gene expression analysis between endotypes

**Figure S6.** Consensus matrices, cluster consensus values, and CDF plot according to the number of clusters in the validation dataset

**SUPPLEMENTARY METHODS**

**Study design and analytic workflow**

We conducted a meta-analysis using publicly available RNA-seq datasets to identify endotypes in sepsis. The analytic workflow is summarized in **Figure S1**. Briefly, we first performed a systematic search to identify eligible RNA-seq data of sepsis. To ensure robust analysis, we preprocessed the data by excluding low-quality samples (e.g., those with low mapping rates), filtering out low-expression genes, performing normalization, and correcting for batch effects across datasets. Second, we applied an unsupervised clustering approach to preprocessed RNA-seq data to identify distinct molecular endotypes. Third, to interpret the biological significance of the endotypes, we performed differential expression gene analysis, pathway enrichment analysis, and immune cell deconvolution analysis. Fourth, we investigated the clinical characteristics and mortality associated with each endotype to assess their potential clinical relevance. Finally, we constructed a multiclass regression model with LASSO regularization to identify gene classifiers for endotype determination. To validate the robustness of our findings, we applied these classifiers to an independent external cohort and assessed the reproducibility of the endotypes and their associated characteristics.

**Data acquisition**

To systematically acquire mRNA-seq data from patients diagnosed with sepsis in public databases, we searched MEDLINE, Scopus, and Web of Science published before April 1st, 2024. The detailed search strategy can be found in **Table S1**. Two independent reviewers (KM and MK) assessed whether the studies satisfied the inclusion criteria. The inclusion criteria were: 1) adult patients diagnosed with sepsis, 2) mRNA-seq data obtained from human whole blood, and 3) available mortality outcome data. Studies were excluded if they met the following criteria: (1) Meta-analyses, systematic reviews, case reports, and conference abstracts, (2) Studies using microarray or single-cell RNA-seq data, (3) Studies primarily focusing on COVID-19, and (4) Studies using publicly available data. Any disagreements were resolved through discussion between the reviewers. From the identified studies, we included data that met the following criteria: (1) Patients meeting Sepsis-3 criteria, with three exceptions: (i) for GSE63042 [1] and GSE131411 [2] using Sepsis-2 definitions, we included patients with severe sepsis or septic shock to ensure alignment with current Sepsis-3 criteria; (ii) for GSE185263 [3], which included patients with suspected sepsis, we selected only those with sequential organ failure assessment (SOFA) scores ≥ 2 to ensure they met the Sepsis-3 diagnostic criteria; (iii) for GSE222393 [4], which included some patients overlapping with GSE185263, we included only unique patients based on the metadata, and (2) For longitudinal studies, we analyzed only the initial time point data (collected within 24 hours of emergency department [ED] or intensive care unit [ICU] admission). This analysis was exempt from IRB review as it utilized de-identified, publicly available data.

**RNA-seq data preprocessing**

We obtained RNA-seq data in sequence read archive (SRA) format and converted it to FASTQ files using the SRA Toolkit (version 3.2.0) [5]. We used Fastp (version 0.23.2) to remove low-quality reads and trim adapters for quality control of the raw sequencing data [6]. We quantified the processed files using Salmon (version 1.10.3) with the human transcriptome (GRCh38) as a reference, with GC bias correction (-gcBias) and mapping validation (-validateMappings) options [7]. We analyzed the count data using the R *tximport* package (version 1.32.0) to convert transcript levels to gene-level expression [8]. We excluded samples with mapping rates below 60% to ensure high-quality gene expression data [9].

We filtered low-expression genes using the filterByExpr function from the R *edgeR* package (version 4.2.1) [10]. We normalized the filtered count data using the trimmed mean of M-values method and corrected for batch effects among different datasets using the ComBat-seq function from the R *sva* package (version 3.52.0) [11, 12].

**Endotyping with RNA-seq data**

We aimed to identify distinct endotypes based on gene expression without prior assumptions. To achieve this, we computed a distance matrix and derived mutually exclusive clusters using a consensus clustering algorithm in the R *ConsensusClusterPlus* package (version 1.68.0) [13]. The algorithm was run with 100 resampling iterations, using 80% subsampling of samples and 100% of features per iteration, with k-means clustering and Euclidean distance. We determined the optimal number of clusters by evaluating three metrics: consensus matrices, cluster consensus values, and the relative change in area under the cumulative distribution function (CDF) curve.

**Biological significance of endotypes**

To interpret the biological differences between the endotypes, we performed three analyses. First, we conducted differential expression gene (DEG) analysis between the endotypes using the R *limma* package (version 3.60.4) [14]. We defined DEGs as genes with absolute Log_2_ fold change ≥ 1 and false discovery rate (FDR) < 0.05. Second, we performed Gene Set Enrichment Analysis (GSEA) using the R *fgsea* package (version 1.30.0) based on the Molecular Signatures Database (version 7.5.1) Hallmark and Gene Ontology biological process gene sets, with gene set size constraints of 15-500 genes for Hallmark analysis and 100-500 genes for GO biological process (BP) ontology [15–17]. Third, we estimated the proportions of immune cells in each sample using the CIBERSORTx [18]. We input transcripts per million-normalized gene expression data and calculated immune cell abundance using the LM22 signature matrix [19], which quantifies 22 types of immune cells. For the analysis, we used only the following parameters set: B-mode batch correction and 100 permutations for significance analysis.

**Mortality of endotypes**

To determine the association of the endotypes with mortality, we constructed an unadjusted logistic regression model.

**Identifying endotype classifier and validation in an external dataset**

To identify a gene classifier for endotype determination, we constructed a multiclass regression model with the least absolute shrinkage and selection operator (LASSO) regularization using the R *caret* package (version 6.0-94). We applied 5-fold cross-validation to minimize potential overfitting and selected the top 200 DEGs for each cluster comparison based on FDR as input. We determined the gene classifiers based on importance scores in the model. We then validated our endotype classification using an external cohort (GSE236713 [20]) by applying the consensus clustering algorithm to the expression of the gene classifier from microarray data. The microarray data was filtered to include only probes with expression levels above the lower 10% threshold and detected in at least 20% of the samples. The algorithm was run with 100 resampling iterations, 87% sample resampling on each iteration, and 100% feature sampling using PAM method and Euclidean distance due to its enhanced robustness to outliers commonly found in microarray datasets. GSE236713 dataset contains microarray data from adult patients with sepsis. This dataset was chosen because it provides comprehensive patient data including laboratory data and mortality outcomes, allowing us to validate both the biological characteristics and clinical relevance of our identified endotypes. Unlike the discovery cohort, GSE236713 was not identified through our systematic search but was selected based on its availability of detailed clinical data supporting our analysis objectives. We assessed the consistency of the endotypes by comparing their GSEA patterns and clinical characteristics with those of the original cohort.

**Statistical analysis**

We analyzed the data using R version 4.4.1 (R foundation, Vienna, Austria). All P-values were two-tailed, with *p* < 0.05 considered statistically significant. We used chi-square and Kruskal-Wallis tests, as appropriate, to compare clinical characteristics and immune cell proportions between the endotypes. For variables showing significant differences, we conducted pairwise comparisons using Wilcoxon tests with Bonferroni correction for multiple testing. Confidence intervals for median differences were estimated using bootstrapping with 1,000 iterations. We accounted for multiple testing using the Benjamini-Hochberg FDR method to the RNA-seq data, which allows for the interpretation of statistical significance in the context of multiple hypothesis testing [21].

**SUPPLEMENTARY REFERENCES**

1. Tsalik EL, Langley RJ, Dinwiddie DL, Miller NA, Yoo B, van Velkinburgh JC, et al. An integrated transcriptome and expressed variant analysis of sepsis survival and death. Genome Med. 2014;6:111.

2. Braga D, Barcella M, Herpain A, Aletti F, Kistler EB, Bollen Pinto B, et al. A longitudinal study highlights shared aspects of the transcriptomic response to cardiogenic and septic shock. Crit Care. 2019;23:414.

3. Baghela A, Pena OM, Lee AH, Baquir B, Falsafi R, An A, et al. Predicting sepsis severity at first clinical presentation: The role of endotypes and mechanistic signatures. EBioMedicine. 2022;75:103776.

4. An AY, Baghela A, Zhang P, Falsafi R, Lee AH, Trahtemberg U, et al. Persistence is key: unresolved immune dysfunction is lethal in both COVID-19 and non-COVID-19 sepsis. Front Immunol. 2023;14:1254873.

5. Leinonen R, Sugawara H, Shumway M. The Sequence Read Archive. Nucleic Acids Res. 2011;39:D19–21.

6. Chen S, Zhou Y, Chen Y, Gu J. fastp: an ultra-fast all-in-one FASTQ preprocessor. Bioinformatics. 2018;34:i884–90.

7. Patro R, Duggal G, Love MI, Irizarry RA, Kingsford C. Salmon provides fast and bias-aware quantification of transcript expression. Nat Methods. 2017;14:417–9.

8. Soneson C, Love MI, Robinson MD. Differential analyses for RNA-seq: transcript-level estimates improve gene-level inferences. F1000Res. 2015;4:1521.

9. Dobin A, Gingeras TR. Mapping RNA-seq Reads with STAR. Curr Protoc Bioinformatics. 2015;51:11.14.1-11.14.19.

10. Chen Y, Chen L, Lun ATL, Baldoni PL, Smyth GK. edgeR v4: powerful differential analysis of sequencing data with expanded functionality and improved support for small counts and larger datasets. Nucleic Acids Res. 2025;53:gkaf018.

11. Leek JT, Johnson WE, Parker HS, Jaffe AE, Storey JD. The sva package for removing batch effects and other unwanted variation in high-throughput experiments. Bioinformatics. 2012;28:882–3.

12. Zhang Y, Parmigiani G, Johnson WE. ComBat-seq: batch effect adjustment for RNA-seq count data. NAR Genom Bioinform. 2020;2:lqaa078.

13. Wilkerson DM, Hayes ND. ConsensusClusterPlus: a class discovery tool with confidence assessments and item tracking. Bioinformatics. 2010;26:1572–3.

14. Ritchie ME, Phipson B, Wu D, Hu Y, Law CW, Shi W, et al. limma powers differential expression analyses for RNA-sequencing and microarray studies. Nucleic Acids Res. 2015;43:e47.

15. Subramanian A, Tamayo P, Mootha VK, Mukherjee S, Ebert BL, Gillette MA, et al. Gene set enrichment analysis: A knowledge-based approach for interpreting genome-wide expression profiles. Proc Natl Acad Sci U S A. 2005;102:15545–50.

16. Liberzon A, Birger C, Thorvaldsdóttir H, Ghandi M, Mesirov JP, Tamayo P. The Molecular Signatures Database (MSigDB) hallmark gene set collection. Cell Syst. 2015;1:417–25.

17. Korotkevich G, Sukhov V, Budin N, Shpak B, Artyomov MN, Sergushichev A. Fast gene set enrichment analysis. Preprint at https://doi.org/10.1101/060012.

18. Newman AM, Steen CB, Liu CL, Gentles AJ, Chaudhuri AA, Scherer F, et al. Determining cell type abundance and expression from bulk tissues with digital cytometry. Nat Biotechnol. 2019;37:773–82.

19. Newman AM, Liu CL, Green MR, Gentles AJ, Feng W, Xu Y, et al. Robust enumeration of cell subsets from tissue expression profiles. Nat Methods. 2015;12:453–7.

20. Szakmany T, Fitzgerald E, Garlant HN, Whitehouse T, Molnar T, Shah S, et al. The “analysis of gene expression and biomarkers for point-of-care decision support in Sepsis” study; temporal clinical parameter analysis and validation of early diagnostic biomarker signatures for severe inflammation and sepsis-SIRS discrimination. Front Immunol. 2023;14:1308530.

21. Benjamini Y, Hochberg Y. Controlling the False Discovery Rate: A Practical and Powerful Approach to Multiple Testing. J R Stat Soc Series B Stat Methodol. 1995;57:289–300.

**Table S1. Search strategies**

| MEDLINE | #1 sepsis[tiab] OR "septic shock"[tiab] OR sepsis [mesh] OR Shock, Septic [mesh] #2 transcriptom*[tiab] OR mRNA[tiab] OR RNA-seq*[tiab] #3 #1 AND #2 |
| --- | --- |
| Web of Science | TITLE-ABS-KEY (sepsis AND "septic shock")  AND  TITLE-ABS-KEY (transcriptom* OR mRNA OR RNA-seq*) |
| Scopus | #1: (ALL=(sepsis)) OR ALL=(septic shock)  #2: ALL=(transcriptom*)  #3: ALL=(mRNA)  #4: ALL=(RNA-seq*)  #5: #2 OR #3 OR #4 #6: #1 AND #5 |

**Table S2.** **Significantly enriched Hallmark biological pathways across endotypes**

Complete data are provided in the accompanying additional file 1.

* The pathways with FDR < 0.05 are shown.

FDR values less than 0.001 are denoted as < 0.001.

† FDR is calculated based on the *p*-value of the pathway enrichment analysis in each endotype.

Abbreviations: AKT, protein kinase B; DN, down; FDR, false discovery rate; G2M, gap 2/mitosis; IL, interleukin; JAK, Janus kinase; KRAS, Kirsten rat sarcoma viral oncogene homolog; mTOR, mammalian target of rapamycin; MYC, myelocytomatosis oncogene; NES, normalized enrichment score; NFKB, nuclear factor kappa B; PI3K, phosphoinositide 3-kinase; ROS, reactive oxygen species; STAT, signal transducer and activator of transcription; TGF, transforming growth factor; TNFA, tumor necrosis factor alpha; UV, ultraviolet.

**Table S3. Significantly enriched GO biological processes across endotypes**

Complete data are provided in the accompanying additional file 2.
* The pathways shown represent pathways with FDR < 0.05, restricted to a maximum of 300 pathways sorted by FDR.

FDR values less than 0.001 are denoted as < 0.001.

† FDR is calculated based on the *p*-value of the pathway enrichment analysis in each endotype.

Abbreviations: deoxyribonucleic acid; ERBB, erythroblastic leukemia viral oncogene B; ERK, extracellular signal-regulated kinase; G protein, guanine nucleotide-binding protein; GTPase, guanosine triphosphatase; IL, interleukin; JAK, Janus kinase; JNK, c-Jun N-terminal kinase; MAP, mitogen-activated protein; NES, normalized enrichment score; NF-kappaB, nuclear factor kappa B; STAT, signal transducer and activator of transcription; TGF, transforming growth factor; TNF, tumor necrosis factor; TOR, target of rapamycin; UV, ultraviolet.

**Table S4. Pairwise comparisons of immune cells across endotypes**

| **Immune cell** | **Comparison** | **Median difference** | **95% CI** | ***P* value** |
| --- | --- | --- | --- | --- |
| Neutrophils | Inflammatory vs. Coagulopathic | 13.3 | 8.3 to 17.6 | <0.001 |
| Neutrophils | Coagulopathic vs. Adaptive | 9.2 | 3.5 to 19.3 | <0.001 |
| Neutrophils | Inflammatory vs. Adaptive | 22.5 | 17.8 to 31.1 | <0.001 |
| B cells memory | Coagulopathic vs. Inflammatory | 0.7 | 0.2 to 1.3 | <0.001 |
| B cells memory | Adaptive vs. Coagulopathic | 1.0 | 0.3 to 2.2 | 0.07 |
| B cells memory | Adaptive vs. Inflammatory | 1.7 | 1.1 to 2.9 | <0.001 |
| T cells CD8 | Coagulopathic vs. Inflammatory | 2.6 | 1.5 to 4.3 | <0.001 |
| T cells CD8 | Adaptive vs. Coagulopathic | 4.4 | 1.2 to 7.2 | <0.001 |
| T cells CD8 | Adaptive vs. Inflammatory | 7.0 | 5.0 to 9.4 | <0.001 |
| T cells CD4 naive | Coagulopathic vs. Inflammatory | 0.1 | -0.5 to 1.4 | 0.30 |
| T cells CD4 naive | Coagulopathic vs. Adaptive | 3.5 | 2.0 to 4.5 | <0.001 |
| T cells CD4 naive | Inflammatory vs. Adaptive | 3.4 | 1.8 to 3.9 | <0.001 |
| T cells CD4 memory resting | Inflammatory vs. Coagulopathic | 0.0 | 0.0 to 0.0 | 0.049 |
| T cells CD4 memory resting | Adaptive vs. Coagulopathic | 0.0 | 0.0 to 0.7 | <0.001 |
| T cells CD4 memory resting | Adaptive vs. Inflammatory | 0.0 | 0.0 to 0.7 | <0.001 |
| T cells CD4 memory activated | Inflammatory vs. Coagulopathic | 0.2 | -0.4 to 0.7 | 1.00 |
| T cells CD4 memory activated | Adaptive vs. Coagulopathic | 2.3 | 1.4 to 3.1 | <0.001 |
| T cells CD4 memory activated | Adaptive vs. Inflammatory | 2.2 | 1.2 to 2.9 | <0.001 |
| T cells regulatory (Tregs) | Coagulopathic vs. Inflammatory | 1.3 | 0.6 to 1.9 | <0.001 |
| T cells regulatory (Tregs) | Coagulopathic vs. Adaptive | 0.3 | -0.7 to 1.3 | 0.88 |
| T cells regulatory (Tregs) | Adaptive vs. Inflammatory | 1.0 | 0.3 to 1.8 | <0.001 |
| T cells gamma delta | Inflammatory vs. Coagulopathic | 0.9 | 0.2 to 1.4 | 0.003 |
| T cells gamma delta | Coagulopathic vs. Adaptive | 0.4 | 0.0 to 0.9 | 0.86 |
| T cells gamma delta | Inflammatory vs. Adaptive | 1.3 | 0.8 to 1.6 | <0.001 |
| NK cells resting | Coagulopathic vs. Inflammatory | 0.9 | -0.1 to 1.6 | 0.13 |
| NK cells resting | Adaptive vs. Coagulopathic | 4.3 | 2.9 to 6.3 | <0.001 |
| NK cells resting | Adaptive vs. Inflammatory | 5.2 | 3.5 to 6.9 | <0.001 |
| Monocytes | Coagulopathic vs. Inflammatory | 4.2 | 2.7 to 7.1 | <0.001 |
| Monocytes | Coagulopathic vs. Adaptive | 0.4 | -1.6 to 3.8 | 1.00 |
| Monocytes | Adaptive vs. Inflammatory | 3.8 | 1.9 to 5.6 | <0.001 |
| Macrophages M0 | Inflammatory vs. Coagulopathic | 0.1 | -1.4 to 1.5 | 1.00 |
| Macrophages M0 | Coagulopathic vs. Adaptive | 1.5 | 0.4 to 2.4 | <0.001 |
| Macrophages M0 | Inflammatory vs. Adaptive | 1.6 | 0.3 to 2.8 | <0.001 |
| Macrophages M1 | Inflammatory vs. Coagulopathic | 0.0 | 0.0 to 0.0 | 0.41 |
| Macrophages M1 | Adaptive vs. Coagulopathic | 0.0 | 0.0 to 0.0 | <0.001 |
| Macrophages M1 | Adaptive vs. Inflammatory | 0.0 | 0.0 to 0.0 | <0.001 |

Statistical comparisons between endotypes were performed using the Wilcoxon rank-sum test with Bonferroni correction for multiple comparisons. Median differences and 95% confidence intervals (CIs) were estimated using bootstrap resampling with 1,000 iterations.

Abbreviation: CI, confidence interval.

**Table S5. Characteristics of patients in each endotype**

|  | **Coagulopathic**  **endotype (n=83)** | **Inflammatory**  **endotype (n=118)** | **Adaptive**  **endotype (n=79)** | ***P* value** | **Total n**^†^ |
| --- | --- | --- | --- | --- | --- |
| Age (year),  median (IQR) | 68 (56-79)  (n=45) | 66 (57-73)  (n=96) | 56 (37-72)  (n=75) | 0.003 | 216 |
| Male sex,  n (%) | 29 (64)  (n=45) | 56 (58)  (n=96) | 41 (55)  (n=75) | 0.58 | 216 |
| SOFA score,  median (IQR) | 3 (2-4)  (n=42) | 5 (3-10)  (n=86) | 3 (2-5)  (n=71) | < 0.001 | 199 |

† Sample sizes vary across variables due to missing data.

Statistical comparisons between endotypes were performed using chi-square and Kruskal-Wallis tests as appropriate.

Abbreviations: IQR, interquartile range; SOFA, sequential organ failure assessment.

**Table S6. Pairwise comparisons of age and SOFA score across endotypes**

| **Variable** | **Comparison** | **Median difference** | **95% CI** | ***P* value** |
| --- | --- | --- | --- | --- |
| Age, (year) | Coagulopathic vs. Adaptive | 12 | 0 to 24 | 0.01 |
| Age, (year) | Coagulopathic vs. Inflammatory | 2 | -8 to 9 | 1.00 |
| Age, (year) | Inflammatory vs. Adaptive | 10 | 2 to 20 | 0.01 |
| SOFA score | Coagulopathic vs. Adaptive | 0 | -1 to 1 | 1.00 |
| SOFA score | Inflammatory vs. Coagulopathic | 2 | 1 to 4 | 0.0002 |
| SOFA score | Inflammatory vs. Adaptive | 2 | 1 to 4 | 0.001 |

Statistical comparisons between endotypes were performed using the Wilcoxon rank-sum test with Bonferroni correction for multiple comparisons. Median differences and 95% confidence intervals (CIs) were estimated using bootstrap resampling with 1,000 iterations.

Abbreviations: CI, confidence interval; SOFA, sequential organ failure assessment.

**Table S7. Endotype assignment matrix and gene importance scores for endotype classification**

| **A** |  |  | | |
| --- | --- | --- | --- | --- |
|  |  | **Original** | | |
|  |  | Coagulopathic  endotype | Inflammatory  endotype | Adaptive  endotype |
| **Predicted** | Coagulopathic  endotype | 75 | 4 | 2 |
|  | Inflammatory  endotype | 8 | 113 | 1 |
|  | Adaptive  endotype | 0 | 1 | 76 |

**B**

| **Gene** | **Importance** |
| --- | --- |
| UGT1A1 | 0.592 |
| NARF | 0.345 |
| SIRPB2 | 0.339 |
| KCNE1 | 0.232 |
| RAB27A | 0.232 |
| LRPPRC | 0.213 |
| TSPYL4 | 0.204 |
| WDR26 | 0.199 |
| ZNF92 | 0.187 |
| PKN1 | 0.171 |
| PYGL | 0.167 |
| HACD3 | 0.166 |
| HLX | 0.164 |
| CHMP2A | 0.146 |

**(A) Endotype assignment matrix:** The endotype assignment matrix demonstrates endotype assignments between original clustering (columns) based on consensus clustering of RNA-seq data from multi-cohorts and predicted classifications (rows) using the 14-gene classifier model. Numbers in cells indicate the count of patients in each category. The diagonal cells (75, 113, and 76) represent concordant assignments between the two methods.

**(B) Gene importance scores for endotype classification:** Relative importance of genes was identified through a multiclass regression model with LASSO regularization.

Abbreviation: LASSO, least absolute shrinkage and selection operator.

**Table S8. Significantly enriched biological pathways based on MSigDB Hallmark gene sets in the validation dataset**

Complete data is provided in the accompanying additional file 3.

* The pathways with FDR < 0.05 are shown.

FDR values less than 0.001 are denoted as < 0.001.

† FDR is calculated based on the *p*-value of the pathway enrichment analysis in each endotype.

Abbreviations: AKT, protein kinase B; FDR, false discovery rate; G2M, gap 2/mitosis; IL, interleukin; JAK, Janus kinase; KRAS, kristen rat sarcoma viral oncogene homolog; mTORC, mammalian target of rapamycin complex; MYC, myelocytomatosis oncogene; NES, normalized enrichment score; NFKB, nuclear factor kappa B; PI3K, phosphoinositide 3-kinase; STAT, signal transducer and activator of transcription; TNFA, tumor necrosis factor alpha; UV, ultraviolet.

**Table S9. Significantly enriched biological pathways based on MSigDB Gene Ontology biological process gene sets in the validation dataset**

Complete data is provided in the accompanying additional file 4.

* The pathways with FDR < 0.05 are shown.

FDR values less than 0.001 are denoted as < 0.001.

† FDR is calculated based on the *p*-value of the pathway enrichment analysis in each endotype.

Abbreviations: FDR, false discovery rate; JNK, c-Jun N-terminal kinase; NES, normalized enrichment score; NF-kappaB, nuclear factor kappa B; TOR, target of rapamycin.

**Table S10. Association of endotypes with mortality in the validation cohort**

|  | Number of deaths, n (%) | Total patients, n | Odds ratio  (95% CI) | *P* value |
| --- | --- | --- | --- | --- |
| Adaptive endotype | 7 (18) | 39 | 1.00 (reference) | – |
| Inflammatory endotype | 8 (20) | 40 | 1.14 (0.37-3.62) | 0.82 |
| Coagulopathic endotype | 15 (34) | 44 | 2.36 (0.87-6.95) | 0.10 |

To investigate the association of endotypes (Adaptive endotype as the reference) with mortality in the validation cohort, logistic regression model was constructed.

Abbreviation: CI, confidence interval.

**Table S11. List of tools and packages used in this analysis**

Complete data is provided in the accompanying additional file 5.

**Figure S1. Study design and analytical workflow for sepsis endotyping**





**1. Data acquisition and preprocessing:** Through systematic screening of publicly available mRNA-seq data, we obtained four datasets of adults with sepsis. After preprocessing, 280 samples met our inclusion criteria and were used for the downstream analyses. **2. Endotyping with mRNA-seq data:** By applying a consensus clustering algorithm to the distance matrix of mRNA-seq data, we identified three distinct endotypes. **3. Biological significance of endotypes:** To interpret the biological significance of the endotypes, we performed differential expression gene analysis, pathway enrichment analysis, and immune cell deconvolution analysis. **4. Clinical characteristics and mortality of endotypes:** We compared clinical characteristics across the identified endotypes. Additionally, we constructed a logistic regression model to investigate the association of the endotypes with mortality. **5. Identifying endotype classifier and validation in an external dataset:** To identify gene classifiers for endotype determination, we constructed a multiclass regression model with LASSO regularization. We then validated our endotype classification using an external cohort (GSE236713) by applying a consensus clustering algorithm to the classifier genes' expression. Some components of this figure were created with BioRender.com.

Abbreviation: LASSO, least absolute shrinkage and selection operator.

**Figure S2. Principal component analysis plots before and after batch correction**


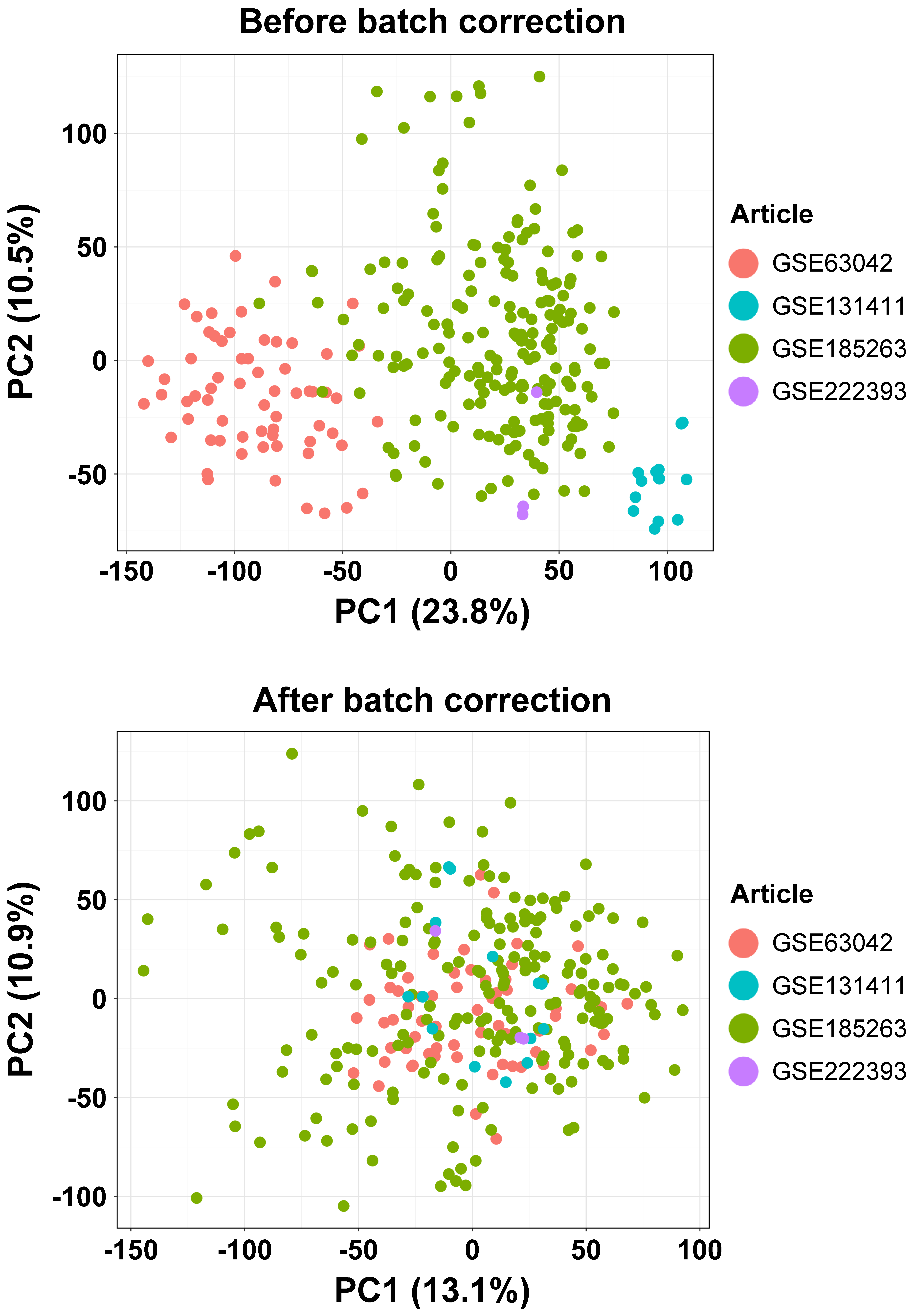


Principal component analysis (PCA) plots show the effect of batch correction on transcriptomic data from four different datasets. The top panel shows the data before batch correction, where samples are clearly clustered by their source dataset, indicating strong batch effects. After batch correction (lower panel), these study-specific clusters are no longer apparent, indicating successful removal of technical variation. The data points are colored by their respective source studies.

Abbreviation: PCA, principal component analysis.

**Figure S3. Consensus matrices, cluster consensus values, and CDF plot according to the number of clusters**


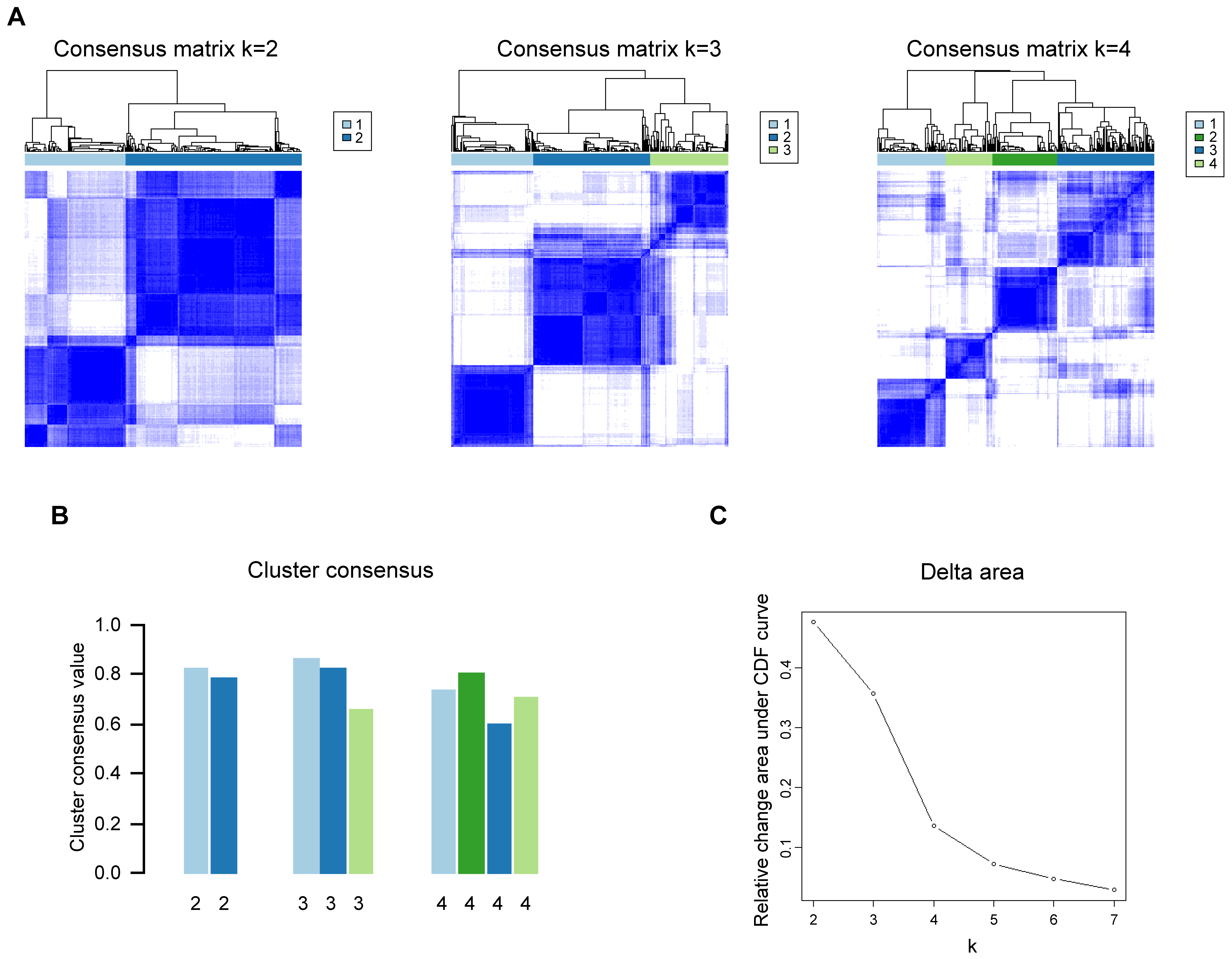


**(A) Consensus matrices**: The heatmaps show the consensus matrices for k=2-4. The color intensity ranges from white to blue, indicating how consistently each sample was assigned to its cluster across multiple iterations. The k=2 and k=3 solutions exhibit clear and stable clustering patterns.

**(B)** **Cluster consensus plot:** The bar plot shows the cluster consensus value of each cluster for different numbers of clusters (k=2-4). High cluster consensus values indicate high stability of the cluster. The cluster consensus values of k=2 and k=3 were high across all clusters.

**(C) Relative change in area under cumulative distribution function curve:** The CDF plot shows the relative change in the area under the CDF curve across the different numbers of clusters (k=2-7). The relative change remained high up to k=3, suggesting that k=3 represents an optimal number of clusters.

Together, these results support the selection of k=3 as the optimal number of clusters for identifying distinct endotypes.

Abbreviation: CDF, cumulative distribution function.

**Figure S4. Immune cell composition of immune cells with lower cell proportion across endotypes**


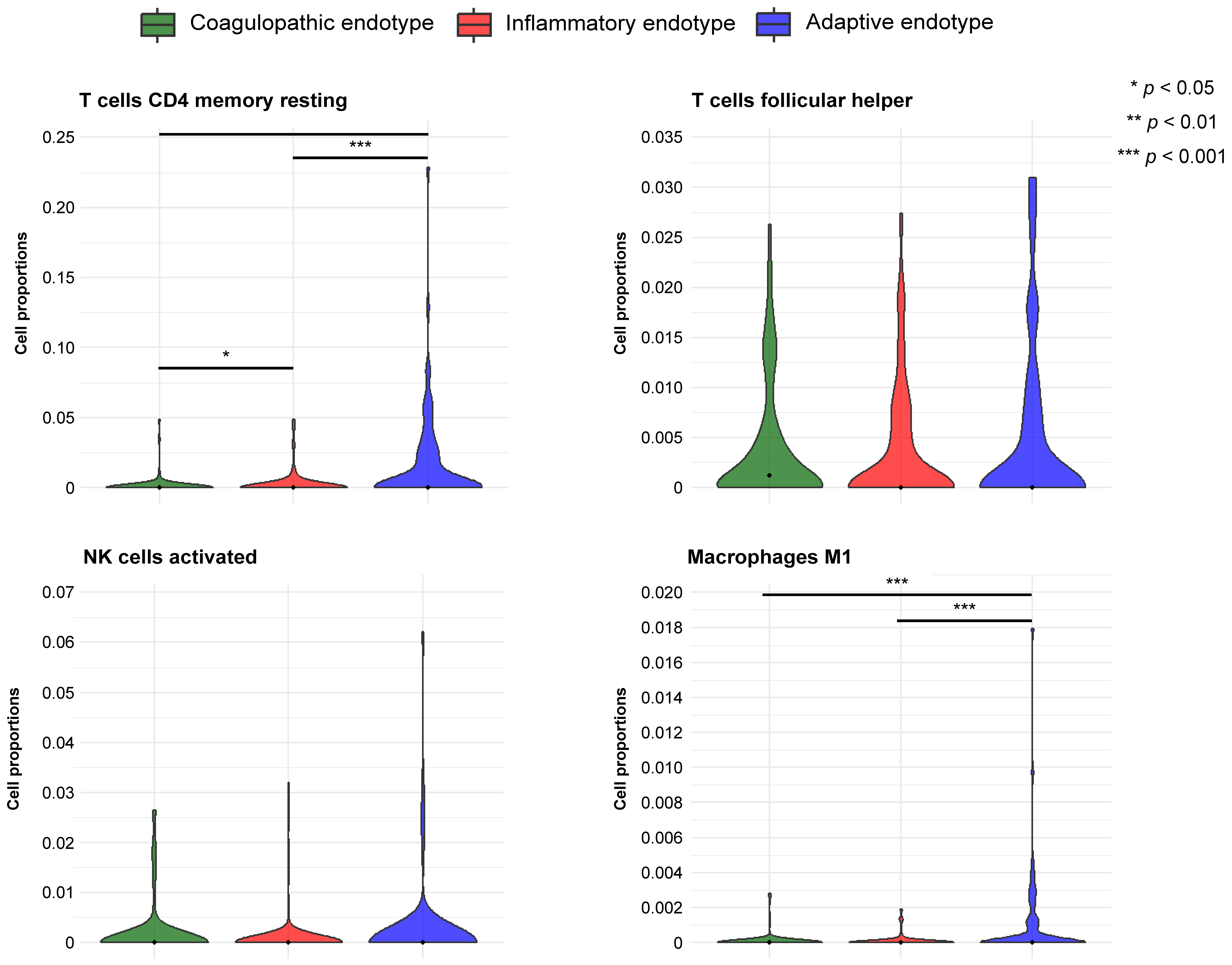


To estimate the proportions of immune cells in each endotype, we used CIBERSORTx. The violin plots represent the distribution of immune cell proportions (y-axis) for each cell type across endotypes. Statistical differences were assessed using Kruskal-Wallis tests followed by Bonferroni-corrected post-hoc tests for cell types showing significant differences. Asterisks indicate statistical significance: ****p* < 0.001, ***p* < 0.01, **p* < 0.05.

Abbreviations: NK, natural killer.

**Figure S5.** **Differential gene expression analysis between endotypes**

**
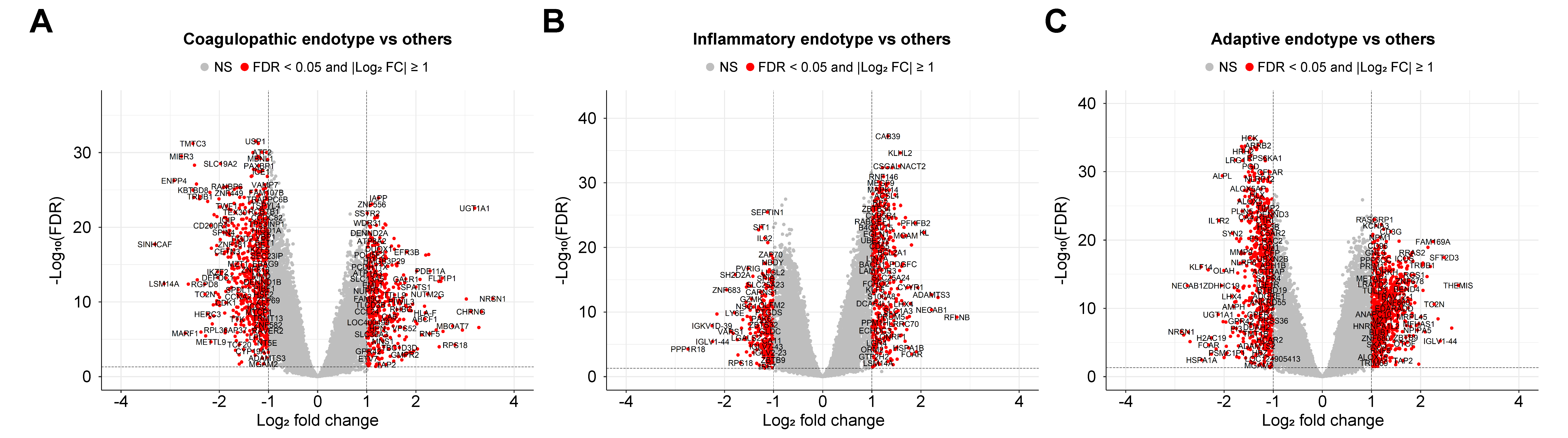
**

The volcano plots show differentially expressed genes between endotypes (A) coagulopathic endotype vs. others, (B) inflammatory endotype vs. others, and (C) adaptive endotype vs. others. Red dots indicate significantly up- and down-regulated genes that meet both fold change (|Log_2_FC| ≥ 1) and statistical significance thresholds (FDR < 0.05). Horizontal dashed lines represent the statistical significance threshold (FDR=0.05), and vertical dashed lines represent the fold change threshold (|Log_2_FC|=1).

Abbreviations: FC, fold change; FDR, false discovery rate; NS, not significance.

**Figure S6. Consensus matrices, cluster consensus values, and CDF plot according to the number of clusters in the validation dataset**


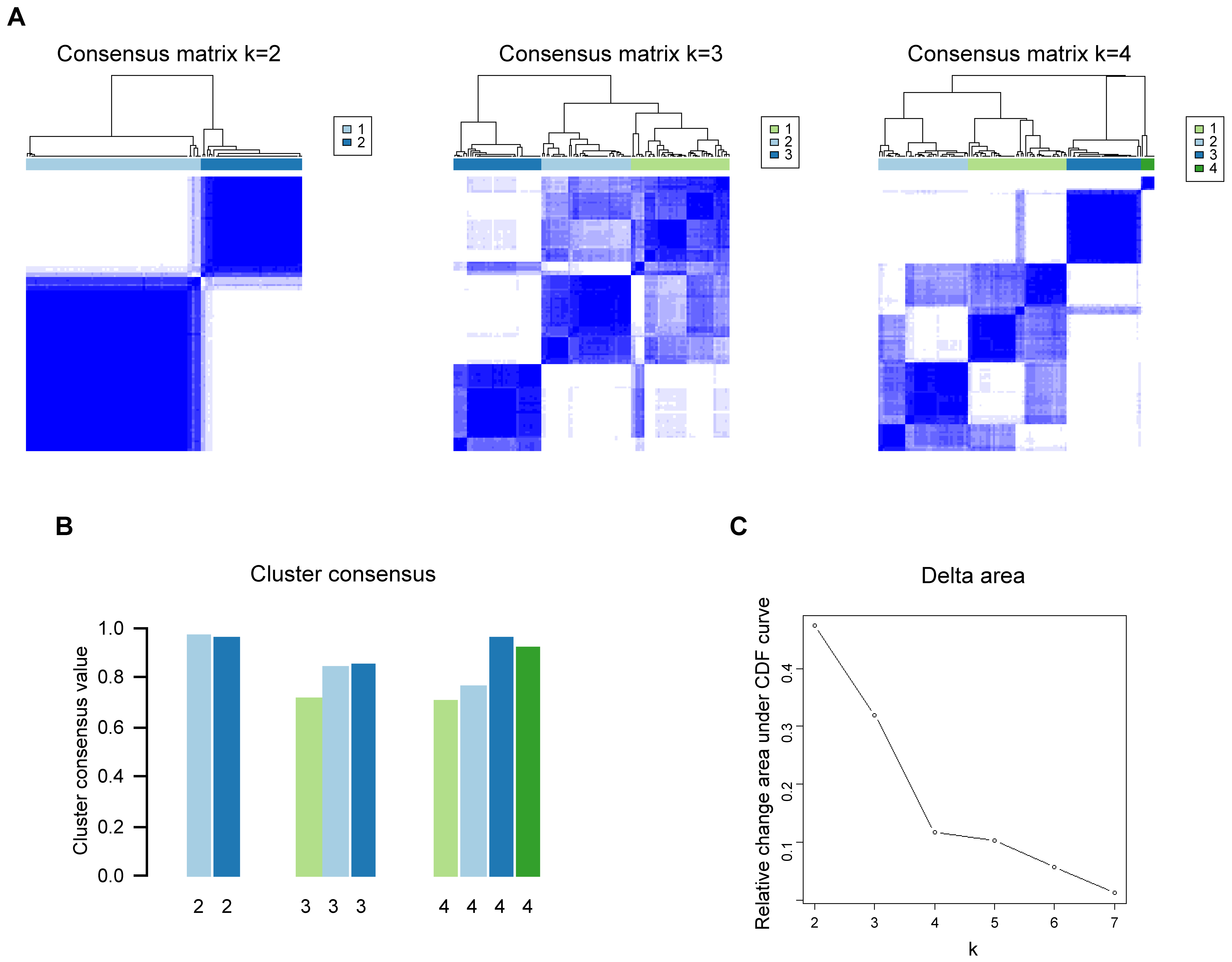


**(A) Consensus matrices**: The heatmaps show the consensus matrices for k=2-4. The color intensity ranges from white to blue, indicating how consistently each sample was assigned to its cluster across multiple iterations. The k=2 and k=3 solutions exhibit clear and stable clustering patterns.

**(B)** **Cluster consensus plot:** The bar plot shows the cluster consensus value of each cluster for different numbers of clusters (k=2-4). High cluster consensus values indicate high stability of the cluster. The cluster consensus values of k=2 and k=3 were high across all clusters.

**(C) Relative change in area under cumulative distribution function curve:** The CDF plot shows the relative change in the area under the CDF curve across the different numbers of clusters (k=2-7). The relative change remained high up to k=3, suggesting that k=3 represents an optimal number of clusters.

Together, these results support the selection of k=3 as the optimal number of clusters for identifying distinct endotypes.

Abbreviation: CDF, cumulative distribution function.
